# Morpho-physiological mechanisms of two different quinoa ecotypes to resist salt stress

**DOI:** 10.1186/s12870-023-04342-4

**Published:** 2023-07-31

**Authors:** Sayed A. Hussin, Safwat Hassan Ali, Muhammad E. Lotfy, Emad H. Abd El-Samad, Mohamed A. Eid, Ali M. Abd-Elkader, Sayed Said Eisa

**Affiliations:** 1 Agricultural Botany Department, Faculty of Agriculture, Ain Shams University, Hadayek Shubra 11241, P.O. Box 68, Cairo, Egypt; 2 Agricultural Biochemistry Department, Faculty of Agriculture, Ain Shams University, Hadayek Shubra 11241, P.O. Box 68, Cairo, Egypt; 3 Vegetable Research Department, Agricultural & Biological Research Institute, National Research Centre, 33 El-Buhouth St, Dokki, 12622 Giza Egypt; 4 Soil Science Department, Faculty of Agriculture, Ain Shams University, Hadayek Shubra 11241, P.O. Box 68, Cairo, Egypt

**Keywords:** Salinity, Quinoa cultivars, Photosynthesis, Osmotic potential, K^+^/Na^+^ ratio

## Abstract

**Background:**

Quinoa (*Chenopodium quinoa* Willd.) is a facultative halophyte showing various mechanisms of salt resistance among different ecotype cultivars. This study aimed to determine salt resistance limits for a Peruvian sea level ecotype “*Hualhuas*” and a Bolivian salar ecotype “*Real*” and elucidate individual mechanisms conferring differences in salt resistance between these cultivars. The plants were grown in sandy soil and irrigated with various saline solutions concentrations (0, 100, 200, 300, 400, and 500 mM NaCl) under controlled conditions.

**Results:**

High salinity treatment (500 mM NaCl) reduced the plant growth by 80% and 87% in *Hualhuas* and *Real* cultivars, respectively. EC_50_ (water salinity which reduces the maximum yield by 50%) was at a salinity of 300 mM NaCl for *Hualhuas* and between 100 and 200 mM NaCl for *Real* plants. Both cultivars were able to lower the osmotic potential of all organs due to substantial Na^+^ accumulation. However, *Hualhuas* plants exhibited distinctly lower Na^+^ contents and consequently a higher K^+^/Na^+^ ratio compared to *Real* plants, suggesting a more efficient control mechanism for Na^+^ loading and better K^+^ retention in *Hualhuas* plants. Net CO_2_ assimilation rates (*A*_*net*_) were reduced, being only 22.4% and 36.2% of the control values in *Hualhuas* and *Real*, respectively, at the highest salt concentration. At this salinity level, *Hualhuas* plants showed lower stomatal conductance (*g*_*s*_) and transpiration rates (*E*), but higher photosynthetic water use efficiency (*PWUE*), indicative of an efficient control mechanism over the whole gas-exchange machinery.

**Conclusion:**

These results reveal that *Hualhuas* is a promising candidate in terms of salt resistance and biomass production compared to *Real*.

## Background

Water shortage and soil salinity are gaining great attention worldwide, due to their negative impacts on plant growth, crop yield, and thus food security, particularly in arid climates [1]. Nearly 8% of the earth’s surface and more than 30% of the global irrigated lands are salt-affected at significant levels [2]. The widespread soil salinization is becoming more prevalent, especially with the extension of intensive agriculture (to meet the future needs of humans) and the inappropriate use of limited water resources [3]. The problem is expected to get even worse over the next decades on the eve of global climatic changes [4, 5]. In this context, the implementation of adaptive measures to sustain crop productivity in salt-affected and marginal areas is a key priority [6]. Sustainable utilization of halophytes in salt-affected and/or degraded lands using saline water would be a feasible solution that fosters crop productivity in such areas [7, 8]. Among a number of underutilized halophytic species, *Chenopodium quinoa* (Family: *Amaranthaceae*), can be an excellent cash crop with tremendous potentials for marginal and salt-affected areas [9–11]. According to Bazile et al. [12], quinoa domestication is thought to have begun in the Andean region 7000 years ago. It is cultivated in various agro-ecological zones from 5° North Latitude in southern Colombia to 43° South Latitude in the Tenth Region of Chile and the Argentinean Andes, with altitudinal distribution ranges from sea level to 4000 m above sea level [13]. The large geographical distribution of quinoa accompanied by a great genetic diversity led to the identification of five quinoa ecotypes namely: salares (salt flats), highlands, inter-Andean valleys, yungas, and coastal lowlands [14]. Due to the broad diversity of its native habitats, quinoa is characterized by a marked variability of environmental adaptation, specifically to soil salinity [15–20], drought [21–23], frost [24], high solar radiation [25] and temperature [26]. Quinoa grains are rich in a wide range of important minerals (Ca, P, Mg, Fe, and Zn), vitamins (B1, B9, C, and E), oil (containing large amounts of linoleate, linolenate, and natural antioxidants), and protein-containing ample amounts of essential amino acids such as lysine and methionine [27–31]. Its potential as a nutritious and resistant crop was recognized by the Food and Agriculture Organization of the United Nations (FAO), which declared the year 2013 as the International Year of Quinoa [32]. Because of these characteristics, quinoa is thrust into the limelight as a non-conventional cash crop, especially in regions where salinity has been recognized as a major agricultural problem [33]. At present, quinoa is cultivated in more than 50 countries outside its origin, with some reports demonstrating an acceptable adaptation in the United States, Canada, Italy, Morocco, India, Pakistan, and Egypt [12]. Incorporation of this promising species into the Egyptian agricultural production system under non-permissive conditions (salinity) calls, however, for precise knowledge about its performance under salt stress, the limits of salinity resistance, and individual mechanisms enabling the plant to grow in saline habitats [16].

In general, the response of quinoa to salinity is characteristic of facultative halophytes, with plant growth stimulation occurring at low and moderate salinity levels [16, 20, 34]. Previous studies demonstrated the ability of some quinoa genotypes to survive even at seawater salinity (up to 50 dS m^− 1^) [10, 34]. Salt resistance of halophytic species is, in most cases, multi-genic, governed by an array of interconnected physiological, morphological, and biochemical mechanisms operating at cellular, organ, and whole plant levels [35]. These mechanisms are closely related to the four major constraints of salinity on plant growth, i.e., osmotic effects, nutritional imbalance, ion toxicity, and restriction of CO_2_ gas exchange [36, 37]. Quinoa has been reported to exhibit a wide range of salt resistance mechanisms. Regarding adaptation to osmotic stress, quinoa showed a very efficient system to adjust osmotically and to reduce its transpiration to maintain a positive water balance in response to salinity [34, 38]. Salinity resistance in quinoa has been attributed to a delicate balance between osmotic adjustment and ion (Na^+^, K^+^, and Cl^–^) accumulation [15, 16, 20, 34]. Nonetheless, increases in organic osmolytes such as proline, glycine betaine, and soluble sugars have been also reported in quinoa [24, 39, 40].

A thorough review of the literature has shown that quinoa displays a high degree of genetic distancing. Its response to salinity stress is strongly genotype dependent, as revealed by many comparative studies on many different accessions, landraces, and cultivars [10, 15, 38, 41, 42]. As a response to salinity, plants have to control their transpiration through sensitive stomatal closure to avoid water loss [37]. Consequently, apparent assimilation rates decline due to restricted CO_2_ availability for carboxylation reactions (stomatal limitation of photosynthesis) [43]. Leaf gas exchange, stomatal conductance, and transpiration rates have been shown to decrease in quinoa under salinity [16, 20, 23, 44, 45]. The plant’s ability to maintain high CO_2_ assimilation rates at minimum H_2_O loss and energy consumption is crucial for its growth under saline conditions [46]. In many salt-resistant species, including quinoa, stomatal limitation of photosynthesis reduces the transpiration rate, leading to higher photosynthetic water use efficiency [20, 40, 47, 48]. While stomatal limitations of photosynthesis are considered the main cause of reduced photosynthetic rate under mild salinity stress, non-stomatal limitations (metabolic and diffusive impairments) become predominant as salinity stress intensifies [49].

Against this background, the present study was designed to screen and compare the eco-physiological responses of a Peruvian (sea level ecotype) quinoa cultivar “*Hualhuas*” and a Bolivian (salar ecotype) cultivar “*Real*” to water salinity under greenhouse conditions. These cultivars originate from different agroecological zones and are expected to exhibit various levels of adaptability to salt stress. Our intent was to determine the limits of salt resistance and the individual adaptive mechanisms conferring resistance differences in these cultivars. Comparing the responses of these closely related cultivars to saline irrigation may give an opportunity for elucidating the key mechanism(s) involved in salinity resistance in quinoa and open prospects for selecting the most suitable cultivar for comprehensive and commercial field trials under Egyptian conditions.

## Results

### Effect of salinity on plant growth and development

**Fig. 1:**
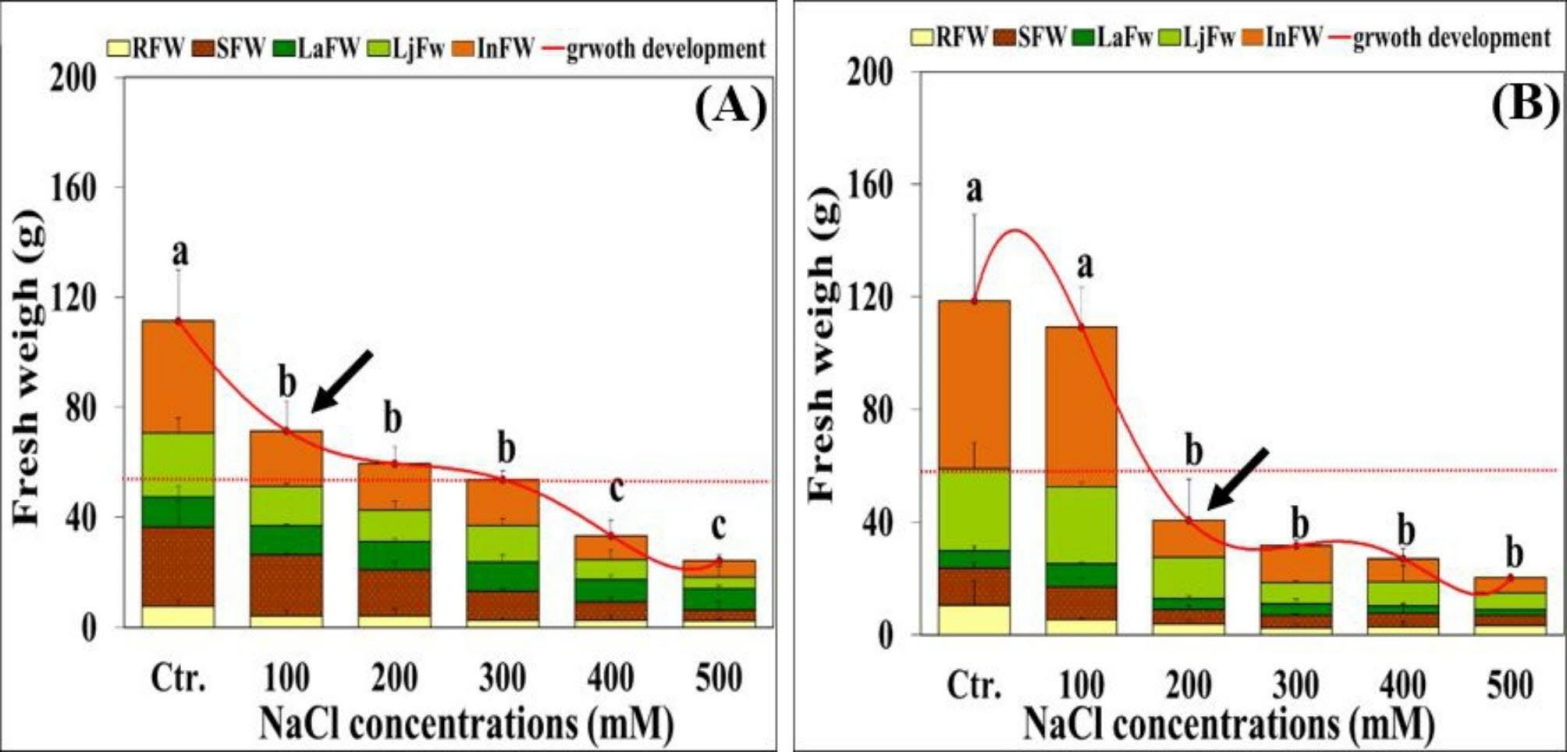
Plant development and growth responses of different organs (expressed as fresh weight) of *C. quinoa* cv. *Hualhuas*** (A)** and *C. quinoa* cv. *Real*** (B)** at various NaCl salinities. The dotted lines mark the EC_50_ values, while arrows show salinity resistance threshold. RFW, root fresh weight; SFW, stem fresh weight; LaFW, adult leaves fresh weight; LjFW, juvenile leaves fresh weight; InFW, inflorescence fresh weight. Each column represents the mean values of six replicates and the bars represent standard errors. Columns with the same letter are not significantly different at *P* < 0.05, according to Duncan’s multiple range test

Comparative growth responses of *Hualhuas* and *Real* plants to varying salinity concentrations are illustrated in Fig. (1A and B). Phenotypic variations between different salinity treatments were visible four weeks after the beginning of salt treatments. Under control conditions, plants of *Real* cultivar showed relatively higher fresh weight (118.5 g/plant) compared to those of *Hualhuas* (112.4 g/plant) (Fig. 1A and B). Increasing NaCl salinity led to a progressive growth inhibition in both cultivars under evaluation, owing to gradual and significant (*P* < 0.05) reductions in the fresh weight of all plant organs (Fig. 1A and B). High salinity treatments (500 mM NaCl) drastically reduced the plant fresh weight by about 80% and 87% in *Hualhuas* and *Real* plants, respectively, relative to the corresponding controls (Fig. 1A and B). In both cultivars, salt-induced growth reduction was much more pronounced for the shoot compared to the root parts, leading to decline the shoot: root fresh weight ratio from 13 to 9 and from 13 to 1 for *Hualhuas* and *Real* plants, respectively. The salt resistance threshold (water salinity that causes initial significant reduction in the maximum expected yield) [50] was at salinity level of 100 mM NaCl for *Hualhuas* plants and at 200 mM NaCl for *Real* plants. EC_50_ was at a salinity of 300 mM NaCl for *Hualhuas* plants and between 100 and 200 mM NaCl for *Real* plants (Fig. 1A and B).

### Effect of salinity on water relations

#### Water content

**Fig. 2:**
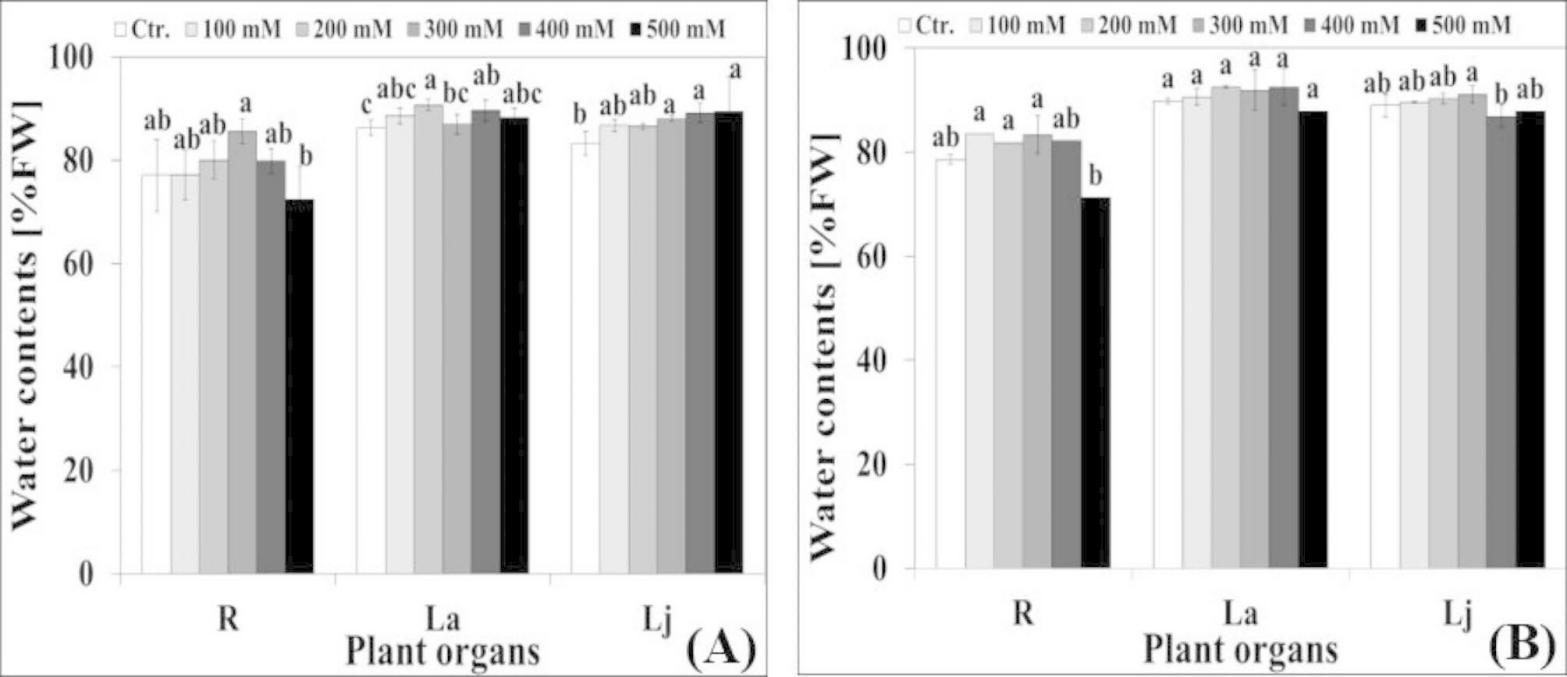
Effect of various NaCl salinity levels on water contents (WC in % FW) of different plant organs of *Hualhuas*** (A)** and *Real*** (B)** plants. R, root; La, adult leaves; Lj, juvenile leaves. Each column represents the mean values of six replicates and the bars represent standard errors. Columns with the same letter are not significantly different at *P* < 0.05, determined by Duncan’s multiple range test

Under control conditions, water content of *Hualhuas* plants was comparatively low, ranging between 77.0% (R) and 86.2% (La). On average over different plant organs, water content of *Real* plants was in the range of 78.58% (R) and 89.8% (La) (Fig. 2A and B). Transient increases in the water contents of all plant organs were observed as NaCl concentration in the external nutrient solution increased (Fig. 2A and B). Maximum water contents, ranging from 85 to 90% and 83–92% for *Hualhuas* and *Real*, respectively, were reached at water salinities between 200 and 400 mM NaCl (Fig. 2A and B). In *Hualhuas* plants, further increase in water salinity slightly reduced the root water content, but increased that of adult and juvenile leaves (Fig. 2A). As for *Real* plants, high salinity treatment slightly reduced the water content of all plant organs relative to their controls (Fig. 2B).

#### Osmotic potential (ψs)

**Fig. 3:**
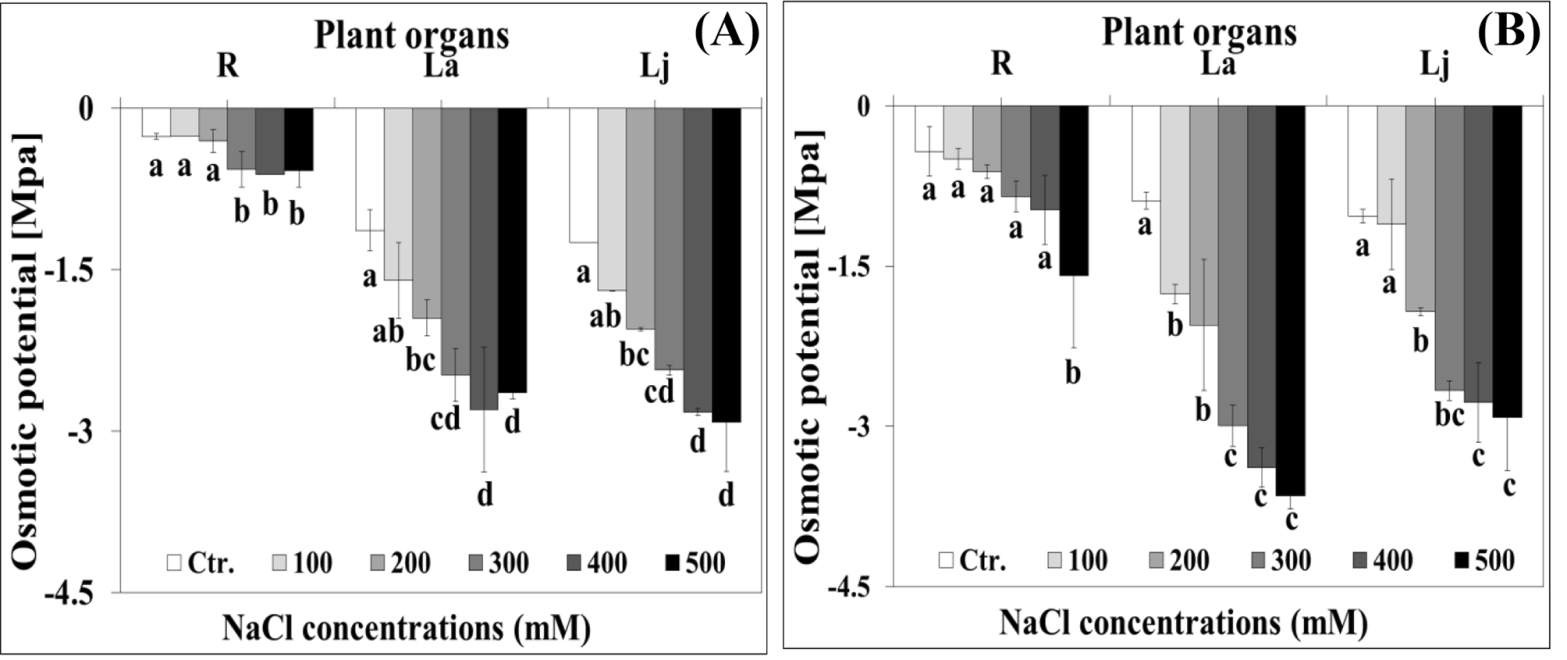
Effect of increasing water salinity on osmotic potential of different plant organs of *Hualhuas*** (A)** and *Real*** (B)** plants. R, root; La, adult leaves; Lj, juvenile leaves. Each column represents the mean values of six replicates and the bars represent standard errors. Columns with the same letter are not significantly different at *P* < 0.05, determined by Duncan’s multiple range test

On average over different plant organs, *ψ*_*s*_ measured from − 0.25 MPa (R) to -1.24 MPa (Lj) and from − 0.42 MPa (R) to -1.03 MPa (Lj) in *Hualhuas* and *Real* plants, respectively, under control conditions (Fig. 3A and B). *ψ*_*s*_ fell gradually in all plant organs and became more negative with increasing water salinity (Fig. 3A and B). It reached from − 0.57 MPa (R) to -2.9 MPa (Lj) and from − 1.59 MPa (R) to -3.69 MPa (La) in *Hualhuas* and *Real* plants, respectively, when the plants were exposed to full strength water salinity (Fig. 3A and B).

### Effect of salinity on Na^+^, K^+^, and K^+^/Na^+^

**Fig. 4:**
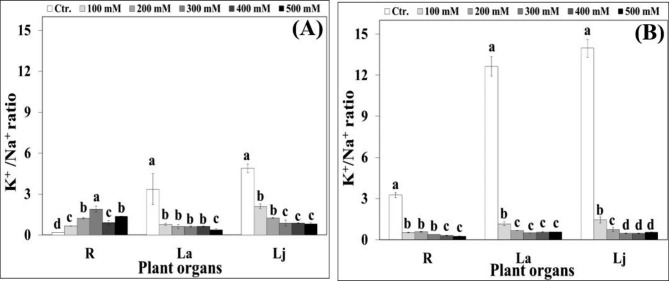
Effect of increasing NaCl salinity on K^+^/Na^+^ ratio of different plant organs of *Hualhuas*** (A)** and *Real*** (B)** plants. R, root; La, adult leaves; Lj, juvenile leaves. Each column represents the mean values of six replicates and the bars represent standard errors. Columns with the same letter are not significantly different at *P* < 0.05, determined by Duncan’s multiple range test

Whatever the salinity treatment, Na^+^ concentrations in roots were lower than those of shoots in both cultivars. Elevating water salinity progressively and significantly increased Na^+^ concentrations, but decreased that of K^+^ in all plant organs of both cultivars under evaluation. This led to a gradual reduction in K^+^/ Na^+^ ratio in both cultivars (Fig. 4A and B). Salt-induced reduction in K^+^/Na^+^ ratio was higher in *Real* plants, as high salinity treatment significantly (*P* < 0.05) declined this ratio by roughly 13, 22, and 25 fold in the roots, adult leaves, and juvenile ones, respectively, compared to the corresponding controls (Fig. 4B).

### Effect of salinity on proline concentration

**Fig. 5:**
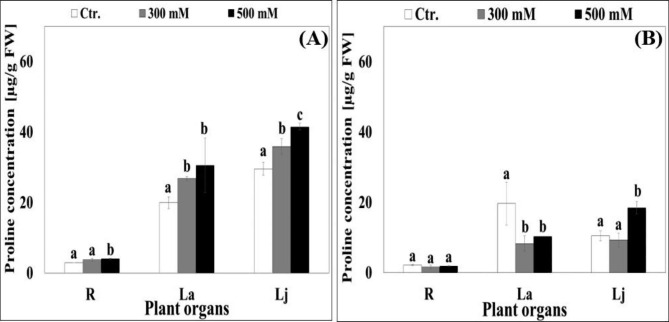
Effect of different water salinity levels on proline concentration (µg g^− 1^ FW) of different plant organs of *Hualhuas*** (A)** and *Real*** (B)** plants. R, root; La, adult leaves; Lj, juvenile leaves. Each column represents the mean values of six replicates and the bars represent standard errors. Columns with the same letter are not significantly different at *P* < 0.05, determined by Duncan’s multiple range test

Under control conditions, low proline concentrations were found in all plant organs, particularly, in the roots of both quinoa cultivars (Fig. 5A and B). On average, proline concentrations were between 2.89 µg g^− 1^ (R) and 29.55 µg g^− 1^ (Lj) in *Hualhuas* plants and between 2.16 µg g^− 1^ (R) and 19.65 µg g^− 1^ (La) in *Real* plants (Fig. 5A and B). In *Hualhuas* plants, proline concentrations enhanced markedly as NaCl salinity rose, with maximal increases of about 40%, 53%, and 40% in roots, adult leaves, and juvenile leaves at seawater salinity (Fig. 5A). The same trend was observed for *Real* plants, but only in the juvenile leaves (75% increases relative to the controls). On the contrary, proline concentrations in the roots and adult leaves were reduced by 12% and 47%, respectively, in this cultivar (Fig. 5B).

### Effect of salinity on photosynthetic pigment and H_2_O/CO_2_-gas exchange parameters

#### Chlorophyll (a), (b), and carotenoid concentrations

**Fig. 6:**
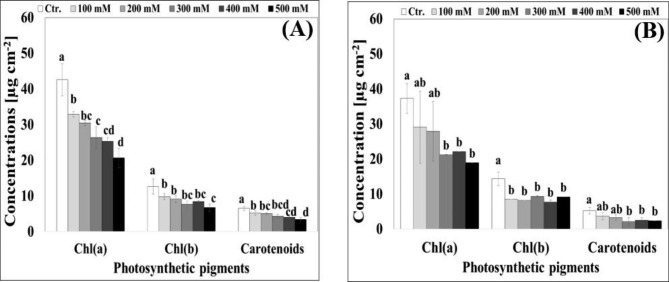
Effect of different water salinity levels on the concentration of photosynthetic pigments of *Hualhuas*** (A)** and *Real*** (B)** plants. Each column represents the mean values of six replicates and the bars represent standard errors. Columns with the same letter are not significantly different at *P* < 0.05, determined by Duncan’s multiple range test

Chlorophyll (a) was the prominent pigment in the leaves of both cultivars, with 42.5 and 38.4 µg cm^− 2^ for *Hualhuas* and *Real* plants, respectively, under control conditions (Fig. 6A and B). It was gradually and significantly (*P* < 0.05) decreased as the external salinity increased. High water salinity led to reductions of 51.4% and 28.6% in *Hualhuas* and *Real* plants, respectively, compared to the respective controls (Fig. 6A and B). The same trend was also observed for Chl (b), as increasing water salinity lowered Chl (b) concentrations in both cultivars, although with a less severe effect compared to Chl (a) (Fig. 6A and B). Full-strength salinity resulted in significant reductions of about 46.7% and 22.4% in *Hualhuas* and *Real*, respectively, compared to controls. As a consequence, the ratio Chl (a)/Chl (b) distinctly declined from 3.3 to 3.0 (*Hualhuas*) and from 3.8 to 3.5 (*Real*) at the highest salinity treatment. Carotenoids concentration was significantly (*P* < 0.05) reduced in the leaves of *Hualhuas* plants, but slightly (statistically not significant) declined in those of *Real* plants in response to water salinity. High water salinity led to reduce their concentrations by about 47.3% and 29.8% in *Hualhuas* and *Real*, respectively (Fig. 6A and B).

#### H_2_O/CO_2_-gas exchange parameters

**Fig. 7:**
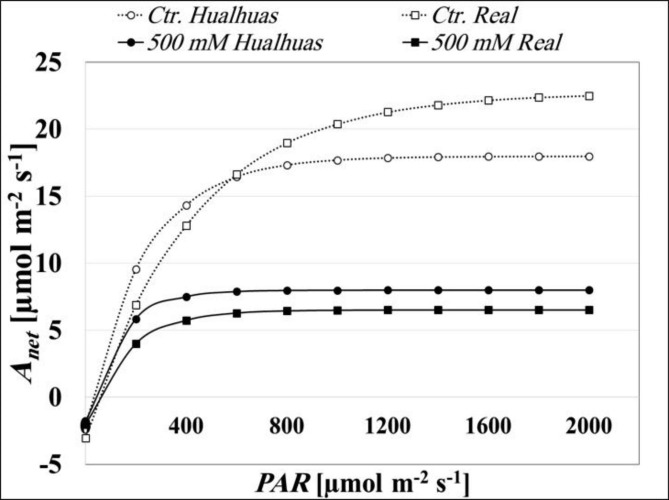
Effect of NaCl salinity on light response curves of *Hualhuas* and *Real* plants. *A*_*net*_, net photosynthetic rate (µmol m^− 2^ s^− 1^); *PAR*, photosynthetic active radiation (µmol m^− 2^ s^− 1^)

The response of CO_2_ assimilation rates (*A*_*net*_) to varying photosynthetic active radiation (*PAR*) is illustrated in Fig. (7). Regardless of salinity treatment, *A*_*net*_ was gradually increased with increasing *PAR*, then started to level off, reached a saturation plateau at *PAR* intensities of 872.3 and 1011.9 µmol m^− 2^s^− 1^ in *Hualhuas* and *Real* plants, respectively, under control conditions (Fig. 7; Table 1). *A*_*net*_ at light saturation measured about 13.5 and 15.1µmol CO_2_ m^− 2^s^− 1^ for *Hualhuas* and *Real* plants, respectively (Table 2). *A*_*net*_ of both cultivars was significantly (*P* < 0.05) and steadily reduced as the water salinity rose, reached only about 22.4 and 36.2 of the control values in *Hualhuas* and *Real*, respectively, at the highest salinity treatment (Table 2). This was accompanied by a decline of about 3.2 and 27.7% in the apparent carboxylation efficiency (*Φ*_*CO2*_) for *Hualhuas* and *Real*, respectively, at the highest water salinity level (Table 1). At this salinity level, the photosynthesis of both cultivars was also saturated at distinctly lower *PAR* intensities compared to respective controls (Fig. 7; Table 1). Additionally, the saturation irradiance (*L*_*s*_) was decreased to 334.5 and 429.0 µmol m^− 2^s^− 1^ in *Hualhuas* and *Real* plants, respectively (Table 1). The light compensation point (*L*_*c*_) was reduced as external salinity rose, being 27.4 and 45.2 µmol m^− 2^ s^− 1^ for *Hualhuas* and *Real* plants, respectively, at full strength salinity (Table 1). Dark respiration (*R*_*d*_) also dropped by 45.3 and 33.4% in *Hualhuas* and *Real* plants at high salinity treatment (Tables 1 and 2). Salt-induced reduction in *A*_*net*_ was accompanied with a gradual and significant (*P* < 0.05) decrease in stomatal conductance (*g*_*s*_). High water salinity treatment led to reduce *g*_*s*_ by about 93.5 and 77.9% in *Hualhuas* and *Real*, respectively (Table 2). This consequently inhibited the transpiration rate (*E*) by 95.3 and 80.2% in *Hualhuas* and *Real*, respectively, at salinity level of 500 mM NaCl (Table 2). In *Hualhuas* plants, the photosynthetic water use efficiency (*PWUE*) was steadily enhanced as the salinity rose, with more than three folds increments at 500 mM NaCl (Table 2). The same trend of salt-induced gradual enhancement in *PWUE* was observed for *Real* plants, but only up to 400 mM NaCl, where *PWUE* was increased by about two folds (Table 2). Higher salinity, however, led to a drastic reduction in *PWUE* by 88.8% in the plants of this cultivar (Table 2). Raising water salinity led to gradual reductions in *C*_*i*_, which consequently lowered the ratio between internal and external CO_2_ concentrations (*C*_*i*_/*C*_*a*_) in *Hualhuas* plants (Table 2). The same tendency was also noted for *Real* plants, but again up to a salinity level of 400 mM NaCl; thereafter, *C*_*i*_/*C*_*a*_ ratio was progressively increased to reach the same levels observed under control conditions (Table 2).

**Table 1:** Leaf gas exchange parameters calculated from *A-PAR* response curves of *Hualhuas* and *Real* plants as affected by water salinity. *Φ*_*CO2*_, apparent carboxylation efficiency (µmol CO_2_ µmol^–1^ photons); *L*_*c*_, light compensation point (µmol photons m^–2^ s^–1^); *L*_*s*_, light saturation point (µmol photons m^–2^ s^–1^); *R*_*d*_, dark respiration rate (µmol CO_2_ m^–2^ s^–1^)

Cultivar	NaCl treatments	***Φ***_***CO2***_ [µmol CO_2_ µmol^− 1^ quantum]		***L***_***c***_ [µmol m^− 2^ s^− 1^]	***L***_***s***_ [µmol m^− 2^ s^− 1^]	***R***_***d***_ [µmol CO_2_ m^− 2^ s^− 1^]	
***Hualhuas***	Ctr.		0.062	49.945	872.297	-3.343	
	500 mM		0.060	27.455	334.467	-1.827	
***Real***	Ctr.		0.054	52.449	1011.860	-3.046	
	500 mM		0.039	45.216	428.980	-2.029	

**Table 2:** Effect of different NaCl salinity treatments on the net assimilation rate (*A*_*net*_), transpiration rate (*E*), photosynthetic water use efficiency (*PWUE*), stomatal conductance (*g*_*s*_) and the ratio between internal and external CO_2_ concentration (*Ci/Ca*) of *Hualhuas* and *Real* plants

Cutivars	NaCl treatments	***A***_***net***_ [µmol m^− 2^ s^− 1^]	***E*** [mmol m^− 2^ s^− 1^]	*PWUE* [*A*/*E*]	***g***_***s***_ [mmol H_2_O m^− 2^ s^− 1^]	***C***_***i***_ ***/C***_***a***_
*Hualhuas*	Ctr.	13.485 ± 0.201^a^	3.144 ± 0.204^a^	4.39 ± 0.023^a^	0.139 ± 0.021^a^	0.743 ± 0.050^a^
	100 mM	10.058 ± 0.705^b^	1.578 ± 0.217^b^	6.85 ± 0.050^b^	0.056 ± 0.010^b^	0.512 ± 0.107^ab^
	200 mM	6.636 ± 0.126^d^	0.767 ± 0.013^c^	8.67 ± 0.018^c^	0.035 ± 0.001^cb^	0.196 ± 0.015^c^
	300 mM	8.076 ± 0.227^c^	0.986 ± 0.003^c^	8.20 ± 0.020^c^	0.047 ± 0.000^b^	0.253 ± 0.017^bc^
	400 mM	3.689 ± 0.209^e^	0.173 ± 0.035^d^	14.14 ± 0.016^d^	0.011 ± 0.002^c^	0.117 ± 0.022^c^
	500 mM	3.016 ± 0.109^e^	0.147 ± 0.027^d^	14.37 ± 0.037^d^	0.009 ± 0.002^c^	0.197 ± 0.035^c^
*Real*	Ctr.	15.120 ± 2.810^a^	3.033 ± 1.170^a^	5.31 ± 0.095^c^	0.195 ± 0.109^a^	0.566 ± 0.112^a^
	100 mM	15.321 ± 3.094^a^	2.834 ± 0.711^a^	5.49 ± 0.055^c^	0.162 ± 0.054^a^	0.534 ± 0.069^a^
	200 mM	7.666 ± 2.794^b^	1.306 ± 0.609^b^	6.10 ± 0.066^c^	0.060 ± 0.032^b^	0.398 ± 0.077^a^
	300 mM	4.487 ± 1.978^ cd^	0.577 ± 0.286^c^	8.38 ± 0.168^b^	0.024b ± 0.012^c^	0.130 ± 0.194^b^
	400 mM	2.449 ± 0.420^d^	0.198 ± 0.020^c^	12.48 ± 0.249^a^	0.008 ± 0.001^c^	0.044 ± 0.006^b^
	500 mM	5.471 ± 3.438^c^	0.598 ± 0.253^c^	0.59 ± 0.045^d^	0.043b ± 0.017^c^	0.593 ± 0.452^a^

Each mean represents six replicates ± standard errors. Means within a column followed by the same letter are not significantly different at *P <* 0.05 as determined by Duncan’s multiple range test

## Discussion

The intention of this study was to determine the range of salt resistance and the individual mechanisms conferring differences in resistance between the Peruvian quinoa cultivar “*Hualhuas*” and the Bolivian cultivar “*Real*”. The growth and biomass accumulation of these cultivars were found to greatly differ depending on salinity level (Fig. 1A and B). Significant differences in plant growth rate, morphological and agronomical traits were previously reported in quinoa and have been attributed to its wide genetic variability [51].

Raising water salinity led to a substantial growth reduction, the response that obviously differed between the quinoa cultivars under evaluation. While low water salinity did not significantly alter the biomass of *Real* plants, it markedly reduced the fresh weight of *Hualhuas* plants. Similarly, Sanchez et al. [44] observed that total plant biomass of *Real* variety was not negatively influenced by water salinity up to 20 ds m^− 1^. Furthermore, several earlier studies reported an optimal growth in *C. quinoa* between 100 and 200 mM NaCl [16, 20]. In accordance with our results, Geissler et al. [47] found that low and moderate NaCl water salinities led to reduce the biomass of *Hualhuas* plants in a pot experiment carried out in an open-top chamber. High water salinity treatment, however, drastically reduced the plant biomass of both cultivars and again, the screened cultivars displayed considerable salt resistance variability. Plants of *Hualhuas* were least affected by high water salinity, exhibiting a growth reduction of about 80% relative to the controls, while plants of *Real* were more sensitive, with biomass reduction of about 87% relative to the controls. Similarly, the growth of the Peruvian cultivar *Hualhuas* [16, 47] and the Danish variety *Titicaca* [34] was significantly inhibited in response to high water salinity (50 ds m^− 1^). Here, the salt resistance threshold was at salinity levels of 10 and 20 ds m^− 1^ for *Hualhuas* and *Real*, respectively, whereas the EC50 was at a salinity of 30 ds m^− 1^ for *Hualhuas* plants and at 10–20 ds m^− 1^ for *Real* plants. Taken together, the relative decline in biomass, salinity resistance threshold, and EC50, indicate that the Peruvian cultivar *Hualhuas* is more salt-resistant compared to the Bolivian cultivar *Real*. In general, high salt resistance requires a root system that is capable to sustain plant growth under stressful conditions. This was indeed the case for *Hualhuas* plants that responded by a general inhibition of the shoot growth, but with continued root growth, resulting in a reduction of the shoot-to-root fresh weight ratio from 13 to 9, compared to 13 to only 1 in *Real* plants. Several factors may act as a bottleneck for plant growth under high salinity [52]. The primary deleterious constraint of salinity on plant growth is due to an osmotic effect [53]. As shown in Fig. (3), *ψ*_*s*_ of all plant organs of both cultivars dropped gradually and became more negative as water salinity rose, the effect that was more obvious for *Real* plants. In accordance with other studies, this implies that quinoa has a very efficient system to adjust osmotically and preserve a positive water balance under saline conditions [16]. This behavior is reflected by the trends of improved water content in all plant organs of both cultivars, particularly under moderate salinities (Fig. 2A and B).

However, high water salinity reduced the water contents of all plant organs of both cultivars, with less severe effects on *Hualhuas* plants. Against these findings and in accordance with Eisa et al. [16], one can presume that osmotic constraint is not a limiting factor for the growth of both cultivars under saline conditions. For both cultivars, salt-induced reduction in *ψ*_*s*_ was associated concurrently with substantial Na^+^ accumulation in all plant organs, particularly in the shoots. This confirms that both cultivars behave as salt-includers, utilizing controlled uptake of inorganic ions to adjust osmotically [16, 19]. Osmotic adjustment by massive accumulation of inorganic ions has been amply reported in many halophytic species, including quinoa [16, 34, 52]. Yet, NaCl salinity progressively increased Na^+^ contents, but significantly decreased those of K^+^ in all plant organs for both quinoa cultivars. This consequently led to a general trend of salt-induced reduction in K^+^/Na^+^ ratio, although with significant differences between cultivars (Fig. 4A and B). This might be due to a competition between K^+^ and Na^+^ uptake or due to changes in membrane integrity caused by the displacement of Ca^2+^ by Na^+^ under saline conditions [54]. In this study, significant differences in Na^+^ accumulation were observed between quinoa cultivars. Plants of *Hualhuas* accumulated distinctly lower amount of Na^+^ in all organs as compared with *Real* plants. This would explain, at least in part, the lower (more negative values) *ψ*_*s*_ observed in the tissues of this cultivar compared to *Hualhuas* (Fig. 3A and B). Another possible explanation for the differences in Na^+^ accumulation could be due to genotypic differences in the rates of Na^+^ uptake and xylem loading between these cultivars, which remains to be elucidated. Salt accumulation in excess of what is required for osmotic adjustment may lead to tissue dehydration, ion imbalance, and/or ion toxicity. Such conditions may result in growth reduction and inhibition of new leaf initiation, and consequently lower salt resistance as observed for *Real* plants. Hence, it is plausible to suggest that *Hualhuas* plants exert more efficient mechanisms to control Na^+^ uptake, translocation, and sequestration at the whole plant level. This is supported by the results of Shabala et al. [11], who found a strong negative correlation between xylem Na^+^ content and salinity resistance and suggested that controlling xylem Na^+^ loading is more important than root Na^+^ exclusion from uptake for salinity resistance in quinoa. At the cellular level, Na^+^ sequestration into the vacuoles is crucial to avoid its toxic effects in the cytosol, while providing a cheap osmoticum for water uptake and turgor maintenance [55]. Nonetheless, ion sequestration by itself is an energy-consuming process (Na^+^ accumulation occurs against a concentration gradient) and is usually accompanied by a concurrent increase in cytosolic osmolality to counteract the high osmolality of vacuoles [52]. This would be achieved by either increasing cytosolic K^+^, or accumulating organic osmolytes (extra energy requirements) in this compartment. As the latter is an energetically expensive option that may cause growth reductions, K^+^ accumulation is much more preferred under high salinity [43, 56]. Reportedly, salinity resistance in quinoa is attributed to its highly efficient K^+^ retention [15, 34, 57]. In the present study, *Hualhuas* plants maintained a higher K^+^/Na^+^ ratio in their organs compared to *Real* plants (Fig. 4). In agreement with Eisa et al. [16], maintaining adequate K^+^/Na^+^ in the tissues of *Hualhuas* plants, especially in the roots and juvenile leaves could be considered as advantageous, insofar, because it means more K^+^ is directed to the most actively growing tissues (where metabolic demands are expected to be greatest and Na^+^ sensitivity is highest). Increases in organic osmolytes have been also reported in quinoa [16, 24, 39]. This is also shown by our data: proline concentration increased by 40–53% in all organs of *Hualhuas* plants (more salt-resistant) in response to salinity (Fig. 5A). The same trend was observed for *Real* (less salt-resistant), but only in the juvenile leaves (75% increases). On contrary, proline concentration in the roots and adult leaves were reduced by 12% and 47%, respectively (Fig. 5B). Ruiz-Carrasco et al. [39] showed that 300 mM NaCl induced proline accumulation in all quinoa accessions tested, the effect that was more pronounced for the most salt-resistant genotypes.

Inadequate cytosolic ion ratios (K^+^/Na^+^, Mg^2+^/Na^+^, and Ca^2+^/Na^+^) can impair the plant photosynthetic machinery. Data of the present study showed that the concentrations of Chl(a) and Chl(b) were gradually and significantly (*P* < 0.05) reduced in both quinoa cultivars with increasing water salinity (Fig. 6A and B). Reduction in chlorophyll concentrations has been widely reported under saline conditions [16, 37] and attributed mainly to ion deficiency, disturbance of chloroplast membranes, instability of protein complexes and enhanced chlorophyllase activity [58, 59]. Our findings also clearly showed that salt-mediated reductions in Chl(a) and Chl(b) were more obvious for *Hualhuas* plants compared to *Real* (Fig. 6A and B). In agreement with Geissler et al. [47], reduction in chlorophyll contents seems to be an adaptive mechanism (from an energetic point of view) to cope with salt stress, since it may lead to reduce the over reduction of the photosynthetic electron transport chain and hence the generation of ROS. Nevertheless, this would lead to the decline of the photosynthetic capacity. As shown in Fig. (7) and Table (2), *A*_*net*_ was greatly decreased in both cultivars as water salinity rose, with more adverse effect on *Hualhuas* plants. Salinity stress led to reduce photosynthesis in a wide variety of halophytic species [37, 60] as well as in several quinoa cultivars [23, 45, 47]. Because plant growth and productivity are inextricably related to its photosynthetic capacity, inhibition of the latter as a response to salinity stress is suggested to be responsible, at least in part, for the reduction in plant growth and yield [52]. In both quinoa cultivars under the study, NaCl salinity affected *A*_*net*_ a priori by enhancing stomatal closure, (stomatal limitation), resulting into substantial reductions in CO_2_ diffusion to the carboxylation sites. This interpretation is supported by the linear proportionality between *A*_*net*_ and *g*_*s*_ (Table 2). Similarly, a positive correlation between the photosynthetic rate and stomatal conductance has been noted in quinoa [10, 16, 47]. Salt-induced reductions in *g*_*s*_ were higher in *Hualhuas* plants (93.5%) when compared to *Real* ones (77.9%) at full-strength salinity treatment (Table 2). Salt-induced inhibition in *g*_*s*_ was accompanied by a progressive decline in *E* (Table 2), likely contributed to a positive water balance. Similar features for water conservation were also reported in quinoa [47] under saline conditions. Salt-induced reduction in *E* was higher in *Hualhuas* plants (Table 2), further suggesting that this cultivar is better adapted to high salinity. Lower *E* can represent an additional adaptive mechanism under high salinity, as it would reduce salt loading into the leaves and hence prolong leaf lifespan by maintaining a subtoxic level of salts [61]. This, indeed, may explain the lower Na^+^ accumulation and thus the higher K^+^/Na^+^ observed for *Hualhuas* plants. As mentioned above, reduced *g*_*s*_ might limit the uptake and diffusion of CO_2_ to the carboxylation sites, as reflected by decreased *C*_*i*_ and hence *C*_*i*_/*C*_*a*_ ratio (Table 2), thus resulting into an impaired *A*_*net*_. The conspicuously low *C*_*i*_ concentration and the linear correlation between *g*_*s*_, *E*, *C*_*i*_, and *A*_*net*_ in *Hualhuas* plants indicate that the limitation of photosynthesis under high salinity conditions in this cultivar is mainly a stomatal one (restricted by stomatal closure and substrate deficiency) [47]. However, this was not the case for *Real* plants, as high salinity led to a marked increase in *C*_*i*_/*C*_*a*_ to reach the control values (Table 2). This suggests that stomatal closure is not a limiting factor for photosynthesis in *Real* under high saline conditions. Impaired photosynthesis in salt-stressed plants can be also due to the leaf biochemical and photochemical (non-stomatal) limitations [16]. This may be due to a decrease in Rubisco activity and/or content, a reduction in RuBP or Pi regeneration, or a decrease in PSII photochemistry efficiency [49]. The latter can decrease light absorption by the leaf [62], which is reflected by lower *L*_*s*_ in both cultivars under saline conditions, particularly, in *Hualhuas* plants (Fig. 7; Table 1). This led, in turn, to a significant reduction (optimization) in *Φ*_*CO2*_ in both cultivars (Table 1). Similar results have been previously reported and interpreted as an important mechanism to reduce the over-reduction of PSII and PSI and hence the generation of reactive oxygen species (ROS) [47]. Salinity-induced reduction in *E* was proportionally higher than that of *A*_*net*_, leading to enhance *PWUE* by more than three folds in *Hualhuas* plants at the highest salinity treatment (Table 2). Such an increase in *PWUE* has been observed for many halophytic species, including quinoa in response to salinity stress [52, 63]. Consistent with previous studies [47, 64], salt-induced improvement in *PWUE* would be an advantage, conferring long-term survival of *Hualhuas* plants under stress conditions. The same trend of an enhanced *PWUE* was also observed for *Real* plants in response to salinity (Table 2), but only up to a salinity level of 400 mM NaCl, thereafter, *PWUE* was drastically decreased by 88.8% in these plants (Table 2). Apparently, this could explain the relatively low salt resistance of *Real* plants compared to *Hualhuas* ones.

## Conclusion

Taken together, our results justified the potential of quinoa as a highly salt-resistant species (in terms of biomass production) able to grow even at 100% seawater salinity (sws). Both quinoa cultivars shared many common features of salt resistance mechanisms, although significant differences in their growth responses were observed. Osmotic constraint was not a major reason for the reduced growth in both cultivars under saline conditions. Rather, salt-induced growth reduction was presumably due to ion deficiency and/or toxicity, leading consequently to an impaired photosynthetic capacity. Results of this study allow for the speculation that the Peruvian cultivar *Hualhuas* is more salt resistant compared to the Bolivian cultivar *Real*. This might be largely attributed to a more efficient control mechanism on xylem Na^+^ loading and better K^+^ retention, ensuring a higher K^+^/Na^+^ ratio compared to *Real* plants. Its lower energy demand and higher responsiveness to balance photosynthesis may also contribute to its higher degree of salt resistance. Finally, it should be mentioned that the Peruvian cultivar *Hualhuas* is not only a promising candidate, suitable for the Egyptian conditions, but also through a deep understanding of its physiological and molecular resistance mechanisms, would provide a possible route to enhance salinity resistance in other genotypes.

## Materials and methods

### Plant materials, experimental design, and growth conditions

The present study was performed at the controlled greenhouse of the Agricultural Botany Department, Faculty of Agriculture, Ain Shams University, Qalyubia Governorate, Egypt (Latitude 30° 06′ 42″ N; Longitude 31° 14′ 46″ E), to investigate the eco-physiological responses of two quinoa cultivars grown under saline conditions. Seeds of *C. quinoa* cv. *Hualhuas* (origin: International Potato Center, CIP, Lima, Peru) and *C. quinoa* cv. *Real* (origin: Salar de Uyuni, Bolivia) were surface-sterilized with 70% ethanol for 1 min and subsequently with 0.5% NaOCl for 3 min before they were rinsed thoroughly with sterile water. The seeds were then sown in black plastic pots (30 cm diameter and 21 cm height), filled with washed sand (8 kg each, on a dry weight basis), five seeds per pot. The pots were kept on a bench at ambient temperatures of 22 ± 3 ºC daytime and 14 ± 3.5 ºC nighttime, a photoperiod of 10 h, relative humidity of 60–70%, and light intensity of 1500–2000 µmol m^− 2^ s^− 1^. The plants were irrigated manually and regularly with a nutrient solution [65]. After the emergence of the first two true leaves (three weeks after the germination), the plants were thinned to two seedlings of uniform size per pot. Salinity treatment started after a period of another two weeks by raising NaCl concentration in the nutrient solution in steps of 100 mM NaCl daily until the final concentrations were achieved to avoid salt shock injuries. There were altogether six salinity treatments (eight replicate pots for each treatment): control, 100, 200, 300, 400, and 500 mM NaCl [equivalent to 0, 20, 40, 60, 80, and 100% seawater salinity (sws)]. Salinity treatments were performed for eight weeks.

### Harvest procedure and growth parameter measurements

The plants were destructively harvested eight weeks after the initiation of salinity treatment (six replicates each treatment). They were separated into roots (R), stems (S), adult leaves (La), juvenile leaves (Lj), and inflorescences (In). The root segments were gently cleaned from sand, washed for 1–2 min with ice-cold distilled water to remove the excess nutrients and salts, and then blotted carefully with tissue paper to remove adhered surface water. The fresh weights of all plant organs were directly recorded. Representative specimens of about 500–1000 mg from each plant organ (R, La, and Lj) were taken and stored at -20 ºC for further quantitative chemical analyses. To obtain the dry weights of different plant organs, specimens of about 500 mg were dried at 70 ºC until they reached a constant weight and the water content was determined as percentages of the fresh weights.

### Determination of osmotic potential

The osmotic potential (*ψ*_*s*_) of the press sap of R, La, and Lj was measured using the freeze-point depression method using an Osmometer (Osmomat 030, Genotec GMBH, Berlin). A 300 mOsmol NaCl solution was used as a standard and the readings were then converted to pressure units using a conversion table according to H-W Koyro [66].

### Determination of mineral elements

Approximately 0.2 g of pulverized dried material from R, La, and Lj were weighed and wet digested using concentrated sulphuric acid (H_2_SO_4_) and hydrogen peroxide (H_2_O_2_ 30%). The cleared, cooled extracts were carefully completed to a final volume of 50 ml with distilled water and then filtered through Whatman filter paper No. 42. Potassium (K^+^) and sodium (Na^+^) concentrations in these extracts were measured using a flame emission photometer method (JENWAY, PFP-7, ELE Instrument Co. Ltd., Essex, UK).

### Photosynthetic pigments and gaseous exchange measurements

#### Determination of photosynthetic pigments

An appropriate amount of fresh materials (ten disks) from the uppermost fully expanded juvenile leaves were extracted in 80% (v/v) aqueous acetone. The concentrations of chlorophyll (a), chlorophyll (b), and carotenoids were determined spectrophotometrically as described by HK Lichtenthaler [67].

#### Leaf gas exchange measurements

The responses of leaf CO_2_/H_2_O gas exchange parameters to different water salinities and light intensities were assessed using an open portable photosynthesis measurement system (LI-COR 6400, Lincoln, NE, USA). One week before harvest, several photosynthetic parameters such as net assimilation rate (*A*_*net*_, µmol CO_2_ m^− 2^s^− 1^), transpiration rate (*E*, mmol H_2_O m^− 2^s^− 1^), stomatal conductance (*g*_*s*_, mmol m^− 2^s^− 1^) and intercellular CO_2_ concentration (*C*_*i*_, µmol mol^− 1^) were determined at various photosynthetic active radiation (*PAR* = 0, 400, 800, 1200, 1600 and 2000 µmol quanta m^− 2^ s^− 1^). These *PAR* values were provided with an artificial LED light source (6400-02B, LI-COR, Lincoln, NE, USA). The relative humidity was maintained at 50–60%, leaf temperature was set at 25 °C, the flow rate was set at 300 µmol s^− 1^, and CO_2_ concentration was maintained at 400 µmol mol^− 1^ inside the leaf chamber. All measurements were achieved between 09:00 and 15:00 o’clock. Assimilation parameters were recorded at each light level following an acclimation period of 5 min and measurements were repeated to obtain, at least six, stable readings for each salinity treatment. Photosynthesis water use efficiency (*PWUE*, defined as the ratio between net assimilation rate and transpiration) was calculated by the LI-6400xtdata analysis program (LI-COR, Lincoln, NE, USA). Values of *A*_*net*_ were plotted against *PAR* and fitted to *A-PAR* response curves with SigmaPlot 12.0 software (Systat Software, Inc.) using an exponential function as explained by Schulte et al. [68]. By means of this function, the initial linear slope of the light response curve, which describes the efficiency of photosynthetic energy conversion in leaves at sub-saturating light intensities (*Φ*_*CO2*_), the light compensation point (*L*_*c*_, the value of *PAR* when *A*_*net*_ = 0), the light saturation point (*L*_*s*_, the value of *PAR* when *A*_*net*_= 90% A_max_) and the dark respiration rate (*R*_*d*_) were calculated.

### Determination of proline

The proline contents of different plant organs (R, La, and Lj) were determined according to the method of Bates et al. [69]. The absorbance of the toluene phase was read using a UV/VIS spectrophotometer (T-60, PG instrument, Wibtoft Leicestershire, UK), at a wavelength of 520 nm, and proline concentration was calculated by comparing sample absorbencies with the standard proline curve.

### Statistical analysis

All data sets were subjected to one-way-ANOVA analysis using the SPSS for Windows statistical data analysis package (SPSS Inc., 2002, release 16, Chicago, Illinois,, USA) in order to determine if significant differences were found among means. To meet all assumptions for ANOVA, data transformation was performed when the original data were not normally distributed. Duncan’s multiple range test was employed to determine if significant (*P* < 0.05) differences occurred between individual treatments.

## Data Availability

The data sets generated during the current study are available from the first author on reasonable request.

## Abbreviations

*A*_*net*_: Net CO_2_ assimilation rate
*C*_*a*_: Ambient CO_2_ concentration
*C*_*i*_: Intercellular CO_2_ concentration
*C*_*i*_*/C*_*a*_: Ratio of intercellular to ambient CO_2_ concentration
*E*: Transpiration rate
EC_50_: Water salinity which reduces the maximum yield by 50%
*g*_*s*_: Stomatal conductance
InFW: Inflorescence fresh weight
*L*_*c*_: Light compensation point
LaFW: Adult leaves fresh weight
LjFW: Juvenile leaves fresh weight
*L*_*s*_: Saturation irradiance
*PAR*: Photosynthetic active radiation
PFW: Plant fresh weight
*PWUE*: Photosynthetic water use efficiency
*R*_*d*_: Dark respiration
RFW: Root fresh weight
ROS: Reactive oxygen species
SFW: Stem fresh weight
sws: Seawater salinity
*Φ*_*CO2*_: Apparent carboxylation efficiency
